# Constitutively active Notch1 signaling promotes endothelial-mesenchymal transition in a conditional transgenic mouse model

**DOI:** 10.3892/ijmm.2014.1818

**Published:** 2014-06-24

**Authors:** JU LIU, FENGYUN DONG, JAMES JEONG, TAKAHIRO MASUDA, CORRINNE G. LOBE

**Affiliations:** 1 Laboratory of Microvascular Medicine, Medical Research Center, Shandong Provincial Qianfoshan Hospital, Shandong University, Jinan, P.R. China; 2 Molecular and Cellular Biology Division, Sunnybrook Health Science Centre, University of Toronto, Toronto, Ontario, Canada; 3 Department of Medical Biophysics, University of Toronto, Toronto, Ontario, Canada

**Keywords:** endothelial-mesenchymal transition, Notch, Cre/loxP, Snail, transgenic mice

## Abstract

Endothelial-mesenchymal transition (EndoMT) is a process in which endothelial cells lose their cell-type-specific characteristics and gain a mesenchymal cell phenotype. The Notch signaling pathway is crucial in the regulation of EndoMT; however, its roles have not been fully studied *in vivo*. In a previous study, we reported the generation of transgenic mice with a floxed β-geo/stop signal between a CMV promoter and the constitutively active intracellular domain of Notch1 (IC-Notch1) linked with a human placental alkaline phosphatase (hPLAP) reporter (ZAP-IC-Notch1). In this study, we examined the results of activating IC-Notch1 in endothelial cells. ZAP-IC-Notch1 mice were crossed with Tie2-Cre mice to activate IC-Notch1 expression specifically in endothelial cells. The ZAP-IC-Notch1/Tie2-Cre double transgenic embryos died at E9.5–10.5 with disruption of vasculature and enlargement of myocardium. VE-cadherin expression was decreased and EphrinB2 expression was increased in the heart of these embryos. Mesenchymal cell marker α-smooth muscle actin (SMA) was expressed in IC-Notch1-expressing endothelial cells. In addition, upregulation of *Snail*, the key effector in mediating EndoMT, was identified in the cardiac cushion of the double transgenic murine embryo heart. The results of the present study demonstrate that constitutively active Notch signaling promotes EndoMT and differentially regulates endothelial/mesenchymal cell markers during cardiac development.

## Introduction

Endothelial-mesenchymal cell transdifferentiation (EndoMT) is the process in which endothelial cells lose their cell- type-specific characteristics and gain a mesenchymal or myofibroblastic phenotype (1). EndoMT may be initiated by cytokines or growth factors secreted by peri-vascular cells (2). The basement membrane under the endothelial cells is likely to be degraded by matrix metalloproteinases (MMPs), then the transitioning endothelial cells become motile and invade the surrounding tissues (3). During EndoMT, endothelial cells lose the expression of their markers, such as vascular endothelial (VE)-cadherin and von Willebrand factor (vWF), and gain the expression of mesenchymal cell markers including vimentin, α-smooth muscle actin (SMA) and type I collagen (1,2,4). EndoMT was first observed in studies on cardiac development (5,6). EndoMT has emerged as a possible mechanism in the pathogenesis of various diseases, including diabetic nephropathy, cardiac fibrosis, intestinal fibrosis, pulmonary hypertension and systemic sclerosis (7–10).

Despite the notable importance of EndoMT for embryonic development and pathologic conditions, the underlying molecular mechanisms involved in EndoMT have yet to be fully elucidated. Substantial evidence has indicated the crucial role of TGF-β signaling in the initiation of EndoMT (11). A number of signaling transduction pathways, including VEGF, NFAT, BMP, Wnt/β-catenin, ErbB, and NF1/Ras, play a role in EndoMT during cardiac development (12). In addition, EndoMT can be modulated in response to manipulations of the Notch pathways in many different endothelial cell types (13). It is also suggested that a number of signaling pathways interact with TGF-β and Notch to mediate EndoMT during heart valve development (14,15).

The Notch signaling pathway is evolutionarily conserved and plays a fundamental role in a number of mechanisms (16,17). Activation of Notch signaling is initiated through ligand-receptor interactions which lead to proteolytic cleavage of the receptor (18). Mammals have four receptors (Notch1, 2, 3, 4) and five ligands (Jagged 1 and 2, and δ-like 1, 3 and 4). Following activation, the intracellular domain of Notch (IC-Notch) translocates into the nucleus and binds the DNA-binding protein CSL (CBF1/suppressor of hairless/Lag-1) through its RAM23 domain (19). The CSL protein (also known as CBF-1/RBP-Jκ) binds to the DNA sequence GTGGGAA in the promoter region of Notch-regulated genes (20). The components of the Notch signaling pathway are crucial for cell fate decisions during morphogenesis and embryonic development (17,21). Notch signaling was thought to be involved in the regulation of vascular smooth muscle differentiation during heart valve and cardiac cushion development (22). Subsequent studies have confirmed the involvement of Notch signaling in the EndoMT process (23–25).

Notch proteins are expressed in most cell types and are involved in a broad spectrum of disorders (26). Studies of the Notch pathway in mice using gain- and loss-of-function approaches have been restricted due to the development of embryonic lethal phenotypes (27–29). To investigate the function of Notch signaling in specific tissues, we established ZAP-IC-Notch1 transgenic mice to take advantage of the Cre recombinase-expressing system to tailor IC-Notch1 expression to particular cell types (30). The ZAP-IC-Notch1 construct utilizes a CMV enhancer-chicken β-actin promoter followed by a loxP-flanked β-geo fusion gene and three polyadenylation (pA) sequences. Downstream of the pA sequence is the coding sequence for the IC-Notch1 protein with an internal ribosomal entry site (IRES)-linked human placental alkaline phosphatase (hPLAP). Therefore, IC-Notch1 is silent in the transgenic mice but can be activated by the introduction of Cre recombinase and excision of the stop signal. IC-Notch1 expression can be monitored by the co-expression with hPLAP following Cre excision.

In this study, ZAP-IC-Notch1 mice were crossed with Tie2-Cre mice, which drive Cre recombination in the entire vascular endothelium (31). The expression of IC-Notch1 was activated in endothelial cells during early development. Constitutively active Notch1 signaling induced disruption of the vasculature, enlargement of myocardium and embryonic lethality at E9.5–10.5. VE-cadherin expression was decreased while EphrinB2 expression was increased. Mesenchymal cell marker α-SMA was expressed in IC-Notch1-expressing cells. In addition, *Snail*, the key effector in mediating EndoMT, was upregulated in the ZAP-IC-Notch1/Tie2-Cre double transgenic mouse embryo heart. Results of this study therefore support the role of Notch signaling in the promotion of EndoMT.

## Materials and methods

### Mice

ZAP-IC-Notch1 transgenic mice were previously generated in our laboratory (30). Tie2-Cre transgenic mice were generously provided by Dr Yanagisawa (University of Texas Southwestern Medical Center, Dallas, TX, USA) (31). The ZAP IC-Notch1 transgene was genotyped by staining ear clips for lacZ expression and by pCCALL PCR using genomic DNA isolated from mouse ear biopsies (32). The Tie2-Cre mice were genotyped by Cre PCR as previously described (31).

### Alkaline phosphatase (AP) staining

The embryos were rinsed in PBS prior to fixing in *lacZ* fix solution (0.2% glutaraldehyde, 50 mM EGTA, pH 7.3, 100 mM MgCl_2_ in 100 mM sodium phosphate, 0.02% NP-40 and 0.01% sodium deoxycholate, pH 7.4) for 5 min. The endogenous APs were inactivated by incubation in PBS at 70–75°C for 30 min. Following washing in AP buffer (100 mM Tris-HCl, pH 9.5, 100 mM NaCl, 10 mM MgCl_2_) for 10 min, the samples were stained with AP staining solution (100 mM Tris-HCl, pH 9.5, 100 mM NaCl, 50 mM MgCl_2_, 0.01% sodium deoxycholate, 0.02% NP-40, 337 mg/ml nitro blue tetrazolium salt (NBT), and 175 mg/ml 5-bromo-4-chloro-3-indolyl phosphate, toluidinium salt (BCIP) (both from Roche Diagnostics, Basel, Switzerland). The staining reaction was allowed to proceed for 10–30 min at room temperature. The samples were then washed extensively in PBS and stored at 4°C. The AP staining on frozen sections was performed based on the same procedure as above except that the sections were counterstained with Nuclear Fast Red (Sigma-Aldrich, St. Louis, MO, USA).

### Immunohistochemistry

Tissue preparation and immunohistochemistry were performed as previously described (33). The primary antibodies used were: anti-platelet endothelial cell adhesion molecule-1 (PECAM-1) monoclonal antibody (1:100; BD Pharmingen, San Diego, CA, USA), anti-SMA antibody (1:200; Sigma-Aldrich), anti-EphrinB2 polyclonal antibody (1:1,000), and anti-*Snail* polyclonal antibody (1:2,000) (both from Santa Cruz Biotechnology, Inc., Santa Cruz, CA, USA). Secondary antibodies were biotinylated rabbit anti-rat (1:500) and goat anti-rabbit (1:200) antibodies from Vector Laboratories, Inc., Burlingame, CA, USA. The peroxidase activities were visualized using streptavidin-horseradish peroxidase (HP) and the diaminobenzidine (DAB) detection system (Vector Laboratories, Inc.). The slides were then counterstained with hematoxylin (Surgipath; Leica Microsystems, Wetzlar, Germany).

### Western blot analysis

The mouse embryo hearts were lysed in ice-cold RIPA buffer (20 mM Tris pH 7.5, 150 mM NaCl, 50 mM NaF, 1% NP-40, 0.1% DOC, 0.1% SDS, 1 mM EDTA and supplemented with 1 mM PMSF and 1 μg/ml leupeptin). The protein concentration was determined using the BCA assay (Bio-Rad, Hercules, CA, USA). Equal amounts of protein were separated by a 10% SDS-PAGE and transferred onto a PVDF membrane. The membranes were blocked with 2.5% BSA, and incubated with the primary antibodies at 4°C overnight in PBS-T. Primary antibodies used were: rabbit anti-VE-cadherin antibody (Abcam, Cambridge, MA, USA), rabbit anti-EphrinB2 antibody, rabbit anti-Snail antibody (both from Santa Cruz Biotechnology, Inc.), and mouse anti-β-actin antibody (Sigma-Aldrich). Immunoreactivity was visualized with HRP-linked secondary antibodies and chemiluminescence.

### Semi-quantitative PCR analysis

Total RNA isolation from mouse embryo hearts was performed using TRIzol reagent (Invitrogen, Carlsbad, CA, USA) according to the manufacturer’s instructions. An aliquot of 2 μg total RNA from each sample was used for the synthesis of cDNA using a High-Capacity cDNA Reverse Transcription kits (Applied Biosystems Inc., Foster City, CA, USA). The cDNA was amplified in a final volume of 20 μl with 1 unit of Taq DNA polymerase (Invitrogen) and 10 pmol of each primer. Oligonucleotide primer sequences are shown in Table I. The PCR products were visualized by ethidium bromide staining following a 1.2% agarose gel electrophoresis.

## Results

### Embryos with endothelial cell-specific expression of IC-Notch1 exhibit disorganized vasculature

**Figure 1:**
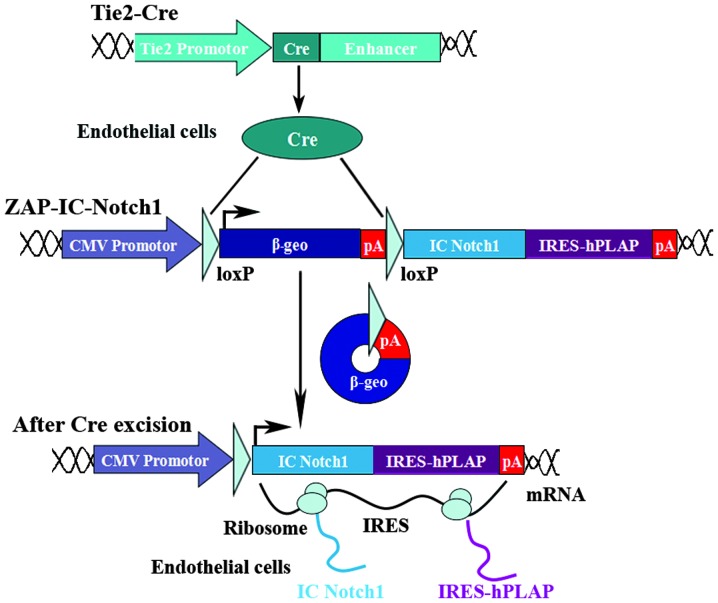
Strategy for Cre-conditional IC-Notch1 expression. The floxed β geo/stop signal was placed between a CMV promoter and the constitutively active intracellular domain of Notch1 (IC-Notch1). The reporter coding sequence of human placental alkaline phosphatase (hPLAP) fused with the internal ribosomal entry site (IRES) was placed downstream of the IC-Notch1 cDNA to allow the co-expression of IC-Notch1 and hPLAP from the same transcript. In the endothelial cells of double transgenic embryos, Tie2-Cre transgene expresses Cre recombinase, which eradicates the β geo/stop signaling sequence. IC-Notch1 expression is then driven by the CMV promoter and detected through visualization of hPLAP.

**Figure 2:**
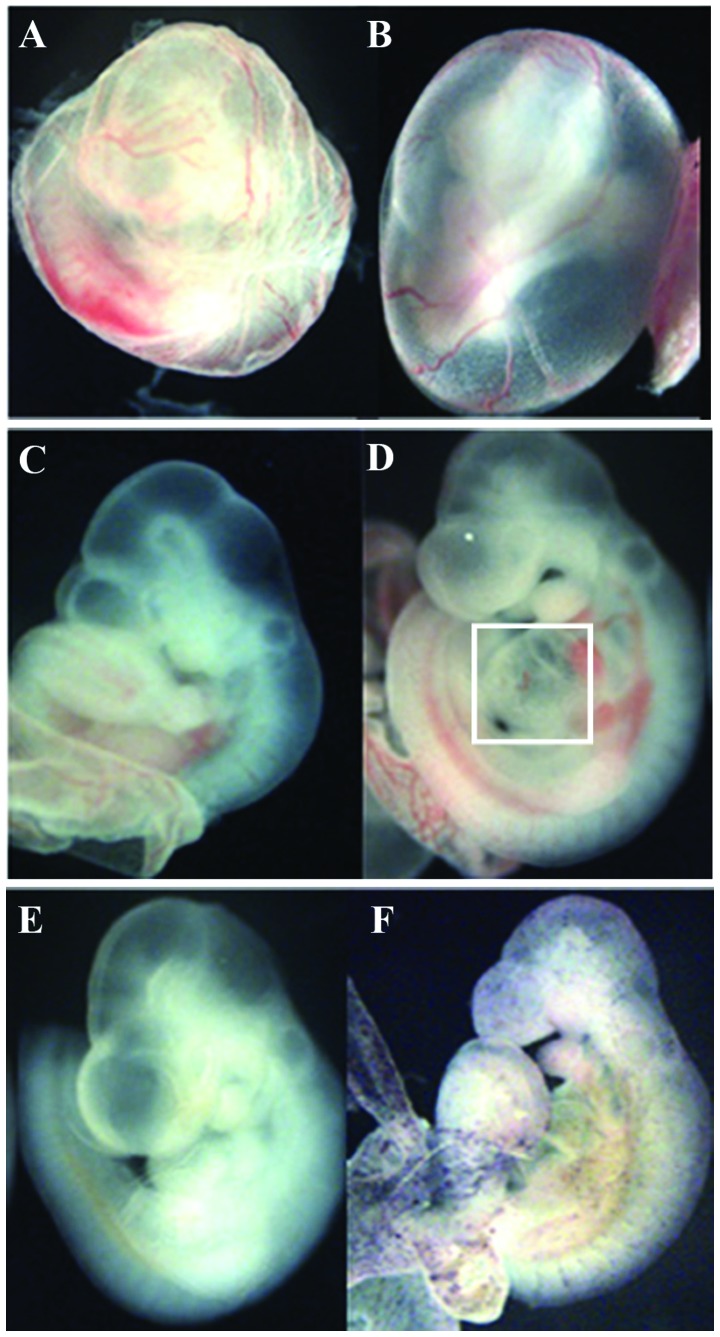
Constitutively active Notch1 signaling in endothelial cells causes defects in cardiovascular development. (A and C) E9.5 wild-type embryo (A) with and (C) without yolk sac. (B and D) E9.5 ZAP-IC-Notch1/Tie2-Cre double transgenic embryo showed (C) disorganized vasculature in the yolk sac and (D) an enlarged heart with hemorrhage in the embryo. The rectangular box refers to the enlarged heart. (E and F) C and D after whole-mount alkaline phosphatase staining.

ZAP-IC-Notch mice were crossed with Tie2-Cre mice to activate IC-Notch1 expression in endothelial cells (Fig. 1). The embryos were taken at various stages, dissected and photographed. The genotypes of the embryos were determined by PCR of yolk sac samples. Double transgenic ZAP-IC-Notch1/Tie2-Cre embryos died at E9.5–10.5 showing pale yolk sacs with fewer blood vessels than the littermates (Fig. 2A and B). These embryos also exhibited an enlarged heart and hemorrhaging around the vessels (Fig. 2D). After whole-mount AP staining, ZAP-IC-Notch1/Tie2-Cre embryos exhibited purple/blue color on the blood vessels in the embryos and yolk sacs (Fig. 2F), indicating that Cre excision of the STOP signal successfully activated constitutive Notch1 signaling together with hPLAP expression.

**Figure 3:**
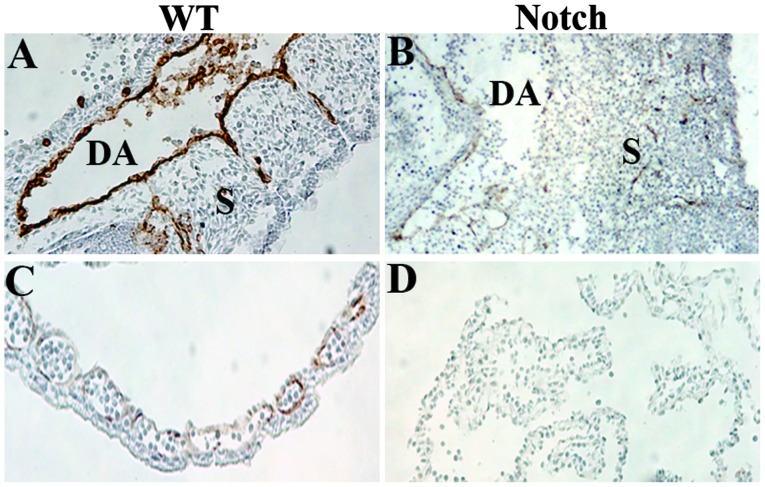
Endothelial-specific expression of intracellular domain of Notch1 (IC-Notch1) disrupts embryonic vasculature. Platelet endothelial cell adhesion molecule-1 (PECAM-1) immunostaining of sagittal sections of (A) normal E9.5 embryo and (C) yolk sac as well as (B) ZAP-IC-Notch1/Tie2-Cre double transgenic embryo and (D) yolk sac. DA, dorsal aorta; S, somite.

The embryos were sectioned and stained with a monoclonal antibody to PECAM-1, a marker for VE cells (34) (Fig. 3). We observed that blood vessels were collapsed in ZAP-IC-Notch1/Tie2-Cre double transgenic embryos, leading to bleeding and the death of the embryos. In the trunk of wild-type embryos at E9.5, intersomitic blood vessels were apparent along the boundaries between adjacent somites (Fig. 3A), but in ZAP-IC-Notch1/Tie2-Cre embryos intersomitic vessels were severely disorganized and irregularly positioned (Figure 3B). Yolk sacs demonstrated the presence of a well-organized capillary bed in wild-type embryos (Fig. 3C), whereas ZAP-IC-Notch1/Tie2-Cre yolk sacs exhibited a disorganized vascular plexus lacking intact blood vessels (Fig. 3D).

### IC-Notch1 promotes cardiac cushion formation and EndoMT in embryo heart

**Figure 4:**
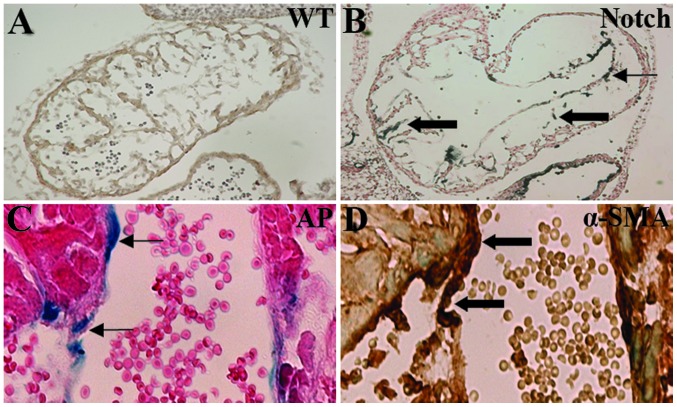
Intracellular domain of Notch1 (IC-Notch1) expression in endocardium promotes endothelial-mesenchymal transition (EndoMT) in the embryo heart. (A and B) Alkaline phosphatase staining of sections of E9.5 wild-type (A) and ZAP-IC-Notch1/Tie2-Cre double transgenic embryo heart (B). Thin arrow refers to positively stained cells on endocardium, thick arrows refer to positive cells in myocardium. (C) Alkaline phosphatase staining of a blood vessel on a section of a ZAP-IC-Notch1/Tie2-Cre double transgenic embryo. Thin arrow refers to positively stained endothelial cells on the blood vessel. (D) α-smooth muscle actin (SMA) immunostaining on the serial section of C. Thick arrow refers to positively stained endothelial cells on the blood vessel.

During cardiac cushion formation from the heart tube, endothelial cells of the endocardium lead to interstitial mesenchymal cells through EndoMT (35). In the ZAP-IC-Notch1/Tie2-Cre mouse embryos, the endocardium of the embryonic heart was intact; however, the cardiac cushion showed hypercellularity and advanced development of heart valves (Fig. 4A and B). On the sections, cells with positive AP staining were observed in the endocardium and myocardium, suggesting that IC-Notch1-expressing endothelial cells migrated into the myocardium (Fig. 4B). α-SMA is expressed in mesenchymal cells such as myofibroblasts, and is not normally expressed in endothelial cells. However, endothelial cells of the ZAP-IC-Notch1/Tie2-Cre mouse embryos, which express IC-Notch1 as shown by AP staining, were stained positive for antibody against α-SMA (Fig. 4C and D). Therefore, these endothelial cells underwent transdifferentiation and gained the characteristics of mesenchymal cells

### IC-Notch1 promotes Snail expression in embryo heart

**Figure 5:**
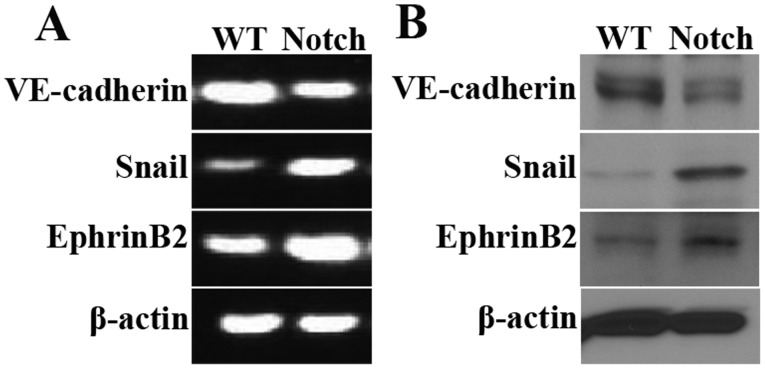
Expression of endothelial-mesenchymal transition (EndoMT)-related proteins in the ZAP-IC-Notch1/Tie2-Cre embryo heart. (A) Semi-quantitative PCR of EndoMT-related genes of RNA extracted from wild-type (WT) and ZAP-IC-Notch1/Tie2-Cre mouse embryo hearts. (B) Representative immunoblots of EndoMT-related proteins examined in protein extracts from WT and ZAP-IC-Notch1/Tie2-Cre mouse embryo hearts.

**Figure 6:**
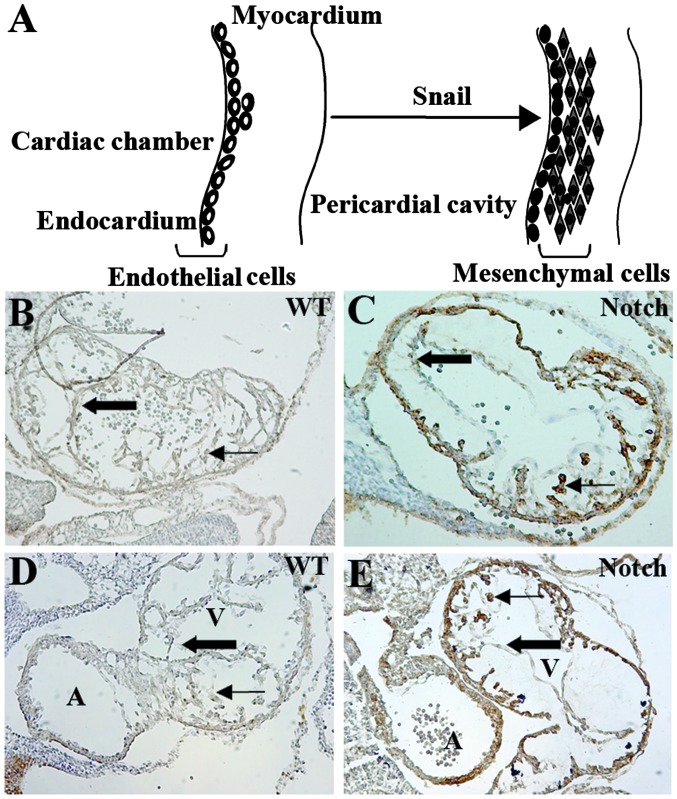
Intracellular domain of Notch1 (IC-Notch1) promotes Snail and EprinB2 expression. (A) Schematic image of endothelial-mesenchymal cell transition. (B-E) Sagittal section of E9.5 embryo heart ventricle: (B) Snail immunostain on wild-type and (C) ZAP-IC-Notch1/Tie2-Cre embryo; (D) EprinB2 immunostain on wild-type and (E) ZAP-IC-Notch1/Tie2-Cre embryo. Dark arrow refers to endocardium, single arrow refers to mesenchymal cells. A, atrium; V, ventricle.

Snail, a Zinc-finger-containing transcriptional repressor, has been identified as a key promoter of EMT (36,37) (Fig. 6A). Results of semi-quantitative PCR and western blot analysis, revealed that the mRNA and protein levels of *Snail* were elevated in the ZAP-IC-Notch1/Tie2-Cre mouse embryo hearts (Fig. 5A and B). The expression of endothelial cell markers was also examined. VE cadherin is an endothelial cell-specific junction molecule and its expression is reduced during EMT. EphrinB2, together with its receptor EphB4, are known to play a crucial role in arteriovenous differentiation (38,39). In the ZAP-IC-Notch1/Tie2-Cre mouse embryo hearts, VE-cadherin expression was decreased while EphrinB2 expression was increased, suggesting that IC-Notch1 differentially regulated endothelial cell markers during EndoMT. Moreover, a strong increase in the Snail protein expression was observed in mesenchymal cells in the cardiac cushion by immunohistochemistry (Fig. 6B and C), and the upregulation of Snail was accompanied by an increase in EphrinB2 expression (Fig. 6D and E).

## Discussion

The Notch signaling pathway is essential for cardiovascular development and is involved in the pathogenesis of many cardiovascular diseases (40). Recently, Notch signaling has been shown to regulate cardiac cushion formation and EndoMT. However, its roles have not been well studied through gain-of-function mouse models. Using the Cre/*loxP* system, we were able to establish transgenic mouse lines carrying a silent transgene for constitutively active IC-Notch1 (ZAP-IC-Notch1). Expression of the IC-Notch1 transgene can be activated in a tissue-specific manner and monitored by an hPLAP reporter. In this study, ZAP-IC-Notch1 mice were crossed with Tie2-Cre mice and constitutively active Notch signaling was triggered specifically in endothelial cells. This unique conditional expression model permitted us to examine the role of Notch signaling in angiogenesis and EndoMT *in vivo*.

During embryonic development, endothelial precursors assemble in a primitive network through vasculogenesis, and the network expands through angiogenesis (41). Angiogenesis is a process of sprouting new capillaries from pre-existing vessels, and is tightly controlled by various cell signaling cascades (42). The Notch signaling pathway plays crucial roles in angiogenesis during embryonic development (21). In mice, the targeted deletion of Notch family genes including Notch receptors or their ligands lead to vascular defects and embryonic lethality at E9.5–10.5 (27–29,43). The gain-of-function studies of Notch signaling are relatively limited. The IC-Notch14 expressed under the *flk-1* locus caused embryonic lethality at E9.5 with a disorganized vascular network (44). In the current study, the Notch signaling pathway was activated specifically in endothelial cells by crossing ZAP-IC-Notch1 mice with Tie2-Cre mice. The double transgenic embryos died before E10.5 with disruption of vasculature in both embryos and yolk sacs. The same outcome was observed for another line of conditional IC-Notch mice ZEG-IC-Notch1 crossed with Tie2-Cre mice (data not shown). The disruption in vasculature produced by constitutively active Notch signaling was similar to the defects in angiogenesis produced by deficient Notch signaling in mouse embryos, suggesting a balance of Notch signaling is required for correct vessel formation during development.

EndoMT is required for embryonic heart development and frequently observed in adult cardiac fibrosis (45). Previous studies have demonstrated an essential role for Notch signaling in the control of endocardial cushion EndoMT (46). In human, mutations of the *Notch1* gene are associated with mitral valve anomalies, bicuspid aortic valve disease and tetralogy of fallot (47). Patients with mutations of the *Jagged1* gene develop Alagille syndrome with cardiac cushion defects (48,49). In mice, the targeted deletion of Notch1 or its key nuclear partner CSL results in cardiac cushion EndoMT defects (50,51). Additionally, the targeted deletion of the downstream Notch effector Hairy/enhancer-of-split associated with the YRPW motif 2 (Hey2) or double-deficiency of Hey1 and Hey2 results in various congenital heart anomalies including cardiac cushion defects (43,52). Notch inhibition in zebrafish embryos similarly prevents cardiac valve development, whereas the transient ectopic expression of activated Notch1 leads to hypercellular valves (51). Similar to zebrafish embryos, results of the present study show that constitutively active Notch1 in endothelial cells increased EndoMT in the mouse embryo heart. In addition, IC-Notch1-expressing cells exhibited the expression of myoblast marker α-SMA and downregulation of the endothelial cell marker VE cadherin. These findings provide further evidence of the involvement of Notch signaling in embryonic development by regulating EndoMT.

The Snail family proteins are zinc finger-containing transcriptional repressors that trigger EndoMT during embryonic development by regulating the expression of junctional proteins such as cadherins (36,53). In mice, *Snail* is expressed in the cardiac cushions after E9.5 (51). Mouse embryos with targeted deletion of Notch1 or CSL lack cardiac *Snail* expression, and show abortive endocardial EndoMT with abnormal maintenance of intercellular adhesion complexes (51). In the embryo heart, Notch functions via lateral induction to promote *Snail*-mediated EndoMT which leads to the cellularization of the developing cardiac valvular primordium (54). In this study, we found that ZAP-IC-Notch1/Tie2-Cre mouse embryo hearts showed a higher expression of *Snail* in the mRNA and protein levels. In addition, an increase in Snail protein expression was observed in mesenchymal cells of cardiac cushion. These findings confirm that Notch signaling promotes *Snail* expression in embryo heart through the gain-of-function mouse model.

VE cadherin is a strictly endothelial-specific adhesion molecule located at junctions between endothelial cells (55). During EndoMT, VE-cadherin expression is reduced in endothelial cells undergoing transdifferentiation (56). The expression of *cadherin 5* gene, which encodes VE-cadherin protein, can be suppressed by Snail transcription factor and Notch signaling components (57). Our results indicate that the mRNA and protein levels of VE cadherin is decreased in ZAP-IC-Notch1/Tie2-Cre mouse embryo heart, which confirms that constitutively active Notch signaling downregulates VE-cadherin expression. EphrinB2 and its receptor EphB4 are involved in determining the boundaries between arteries and veins (39). Although its expression is known to be arterial endothelial cell-specific, EphrinB2 is also expressed in perivascular mesenchymal cells (58). Dll4/Notch1 is upstream of EphB4/ephrinB2 signaling during cardiovascular development (59,60). In this study, EphrinB2 expression was upregulated in ZAP-IC-Notch1/Tie2-Cre mouse embryo heart and immunostaining showed an increased EphrinB2 expression in cardiac mesenchymal cells, suggesting that Notch signaling differentially regulates endothelial cell markers during EndoMT.

In summary, we employed a Cre/loxP conditional mouse model and specifically activated IC-Notch1 expression in endothelial cells. The results demonstrate that constitutively active Notch signaling inhibits angiogenesis and promotes EndoMT in mouse embryos through the gain-of-function mouse model. In addition, IC-Notch1 promotes Snail expression and differentially regulates endothelial/mesenchymal cell markers. The present study provides insights into the role of Notch signaling in EndoMT and cardiovascular development.

## Figures and Tables

**Table I:** PCR primer sequences.

Gene	Sequences	Size (bp)	Tm (°C)
mVE-cadherin	Forward: TCCTCTGCATCCTCACTATCACA	122	60.63
	Reverse: GTAAGTGACCAACTGCTCGTGA		60.55
mSnail			
	Forward: GCCGGAAGCCCAACTATAGCGA	469	64.68
	Reverse: TTCAGAGCGCCCAGGCTGAGGTACT		69.30
mEphrin-B2			
	Forward: CAAGTTCTGCTGGATCAGCCA	124	60.61
	Reverse: TCGGTGCTAGAACCTGGATTT		59.09
β-actin			
	Forward: GGCACCACACCTTCTACAATG	352	59.19
	Reverse: GTGGTGGTGAAGCTGTAGCC		60.96

All sequences have a 5′→3′ orientation. Tm, temperature.
